# Alternative and antioxidant therapies used by a sample of infertile males in Jordan: a cross-sectional survey

**DOI:** 10.1186/1472-6882-14-244

**Published:** 2014-07-16

**Authors:** Sanaa K Bardaweel

**Affiliations:** 1 Department of Pharmaceutical Sciences, Faculty of Pharmacy, The University of Jordan, Queen Rania Street, Amman 11942, Jordan

**Keywords:** CAM, Male infertility, Antioxidants, Medicinal plants

## Abstract

**Background:**

Complementary and alternative medicine (CAM) is frequently used in the Middle East, especially to treat chronic diseases such as infertility. We aimed to examine the prevalence, characteristics, and determinants of CAM use, particularly herbs and antioxidant therapies, among infertile males presenting for infertility evaluation in Jordan.

**Methods:**

Demographic information, use of alternative and antioxidant therapies for infertility treatment, and patients’ belief in efficacy and safety of the therapies used were collected using a face-to-face questionnaire. Data were collected from 428 infertile male patients presenting at infertility clinics in Amman, the capital city of Jordan. The study was conducted between April 2013 and September 2013.

**Results:**

Of the 428 men who completed the questionnaire, 184 (43%) used at least one of the alternative and antioxidant therapies specified in the questionnaire. Nutritional regime; vitamins, such as vitamins C and E; and medicinal herbs, such as ginger, saw palmetto, and ginseng were the most commonly used therapies reported. A correlation between the use of alternative and antioxidant therapies versus infertility duration was found. Additionally, the majority of males using CAM did not inform their health care providers about their usage.

**Conclusions:**

The high prevalence of CAM use among infertile male patients underscores the urge to assimilate CAM into the education and training of health professionals, as well as to improve infertile patients’ knowledge of the safe use of CAM modalities.

## Background

Infertility affects approximately 10–15% of all couples in industrialized countries [1-3]. Approximately 30% to 50% of these cases are owing to male factor infertility [2]. Male factor infertility is defined as a change in sperm concentration, motility, and/or morphology in at least one sample of two sperm analyses collected between 1 and 4 weeks [4].

The generation of oxidants, also known as reactive oxygen species (ROS), in the male reproductive tract is a concern because of their potential toxic effects at high levels on sperm quality and function [5,6]. The oxygen paradox is a challenge faced by all cells living under aerobic conditions, including spermatozoa. While oxygen is required for life, its ROS metabolites endanger cell survival and functions [7]. Male germ cells, at various differentiation stages, generate minute amounts of ROS that are required for sperm production and maturation [8,9]. To preserve normal cell function, excess ROS must be constantly eliminated by seminal plasma antioxidants. Excessive ROS generation may adversely affect sperm number, motility, quality, and function including damage to sperm DNA integrity [10-12]. Several studies have demonstrated that elevated ROS levels are detected in the semen of 25% to 40% of infertile men [7,12,13]. Spermatozoa are intensely vulnerable to the damage provoked by excessive ROS as their plasma membranes enclose considerable amounts of polyunsaturated fatty acids [14] while their cytoplasm has low concentrations of ROS-scavenging enzymes [7].

Enzymatic antioxidants, such as superoxide dismutase, the glutathione peroxidase/glutathione reductase system, and catalase [15], as well as non-enzymatic antioxidants, such as ascorbate (vitamin C) [16], tocopherol (vitamin E) [17], taurine, and hypotaurine [18], are available in the seminal plasma to prevent critical damage provoked by ROS. In fact, the seminal plasma of fertile men has a higher total antioxidant capacity than the seminal plasma of infertile men [19]. However, elevated levels of ROS measured in the semen of infertile men are more likely owing to increased ROS production rather than to reduced antioxidant capacity of the seminal plasma [20].

While the available treatments for male infertility have erratic success rates [21,22], assisted reproductive technologies, such as intrauterine insemination and *in vitro* fertilization, potentially enhance the ability of infertile men to contribute to a pregnancy. For many infertile men, the lack of accessible successful medical therapies has led to the use of alternative therapies, including the use of herbs and antioxidants. The use of alternative therapies in infertile men is usually associated with speculation that some forms of male infertility are caused by antioxidant deficiency. Therefore, the hypothesis is that supplementary antioxidants may enhance their ability to contribute to a pregnancy.

We aimed to gain insights into the patterns of use of alternative and antioxidant therapies in infertile men in Jordan. The prevalence and factors leading to the use of such therapies among infertile men were also investigated. Moreover, one of the objectives of the study was to find out which complementary and alternative medicines (CAM) are being used by infertile men, and whether they believed in the effectiveness of these therapies.

## Methods

### Patient population

Verbal informed consent to participate in the study was obtained based on a standard written statement. Ethical approval for conducting the study was obtained from the Institutional Review Board (IRB) at the Jordan University Hospital (JUH) and the Scientific Committee at the Deanship of Scientific Research at The University of Jordan. Patient information for this study remained confidential and within the institution.

Data were collected from male patients seeking infertility treatment in two types of facilities: *in vitro* fertilization (IVF) centers, at both public and private hospitals, and infertility private clinics. In the private clinics, patients were seen for routine examination and treatment of infertility without any conception assistance being offered. Patients at IVF centers were given conception assistance. Both types of facilities were distributed in different areas of Amman, the capital city of Jordan. The study took place between April 2013 and September 2013. Only male patients attending any of the study centers were invited to complete the questionnaire.

### Questionnaire design

The study questionnaire was structured based on preliminary discussions with patients and health professionals. Additionally, the questionnaire was validated by a committee whose members were health professionals, consisting of two fertility specialists, a pharmacist, and a nutritionist. The questionnaire was written in English and then translated into Arabic. Both versions of the questionnaire were checked by three members of the public with no medical background.

The questionnaire was composed of 18 questions and divided into three sections (Additional file 1). The first section considered patients’ demographic information that may have an impact on their health-related attitudes. In particular, patients were asked about age, education, and income level. In the second section, patients were asked about their medical and infertility history. The third section involved questions about the use of antioxidant therapies, as well as other forms of alternative therapies for their infertility condition. Additionally, patients were asked whether they informed their principal healthcare provider about usage of the reported alternative and antioxidant therapies.

The 18-point anonymous structured questionnaire was given to 428 infertile males presenting at any of the study centers. The study population was then subgrouped into two groups, Group A and Group B. Group A included patients who reported the use of alternative and antioxidant therapies as an infertility treatment aid, and Group B included patients who did not use any form of alternative and antioxidant therapies for their infertility treatment.

### Data analysis

Data were coded, entered, and analyzed using the Statistical Package for Social Sciences program (SPSS) database for Windows, version 17 (SAS Institute, Cary, NC, USA). The analysis of answers involved descriptive quantitative statistics, like frequency and percentage. Chi-square and Fisher exact tests were used to test for significant association between groups. All hypothesis testing was two-sided, with a probability value of 0.05 deemed significant.

## Results

**Figure 1:**
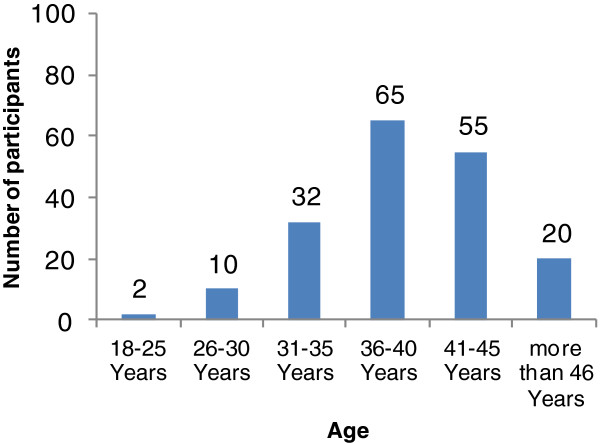
Prevalence of alternative and antioxidant therapies used for infertility treatment among participants by age group.

**Figure 2:**
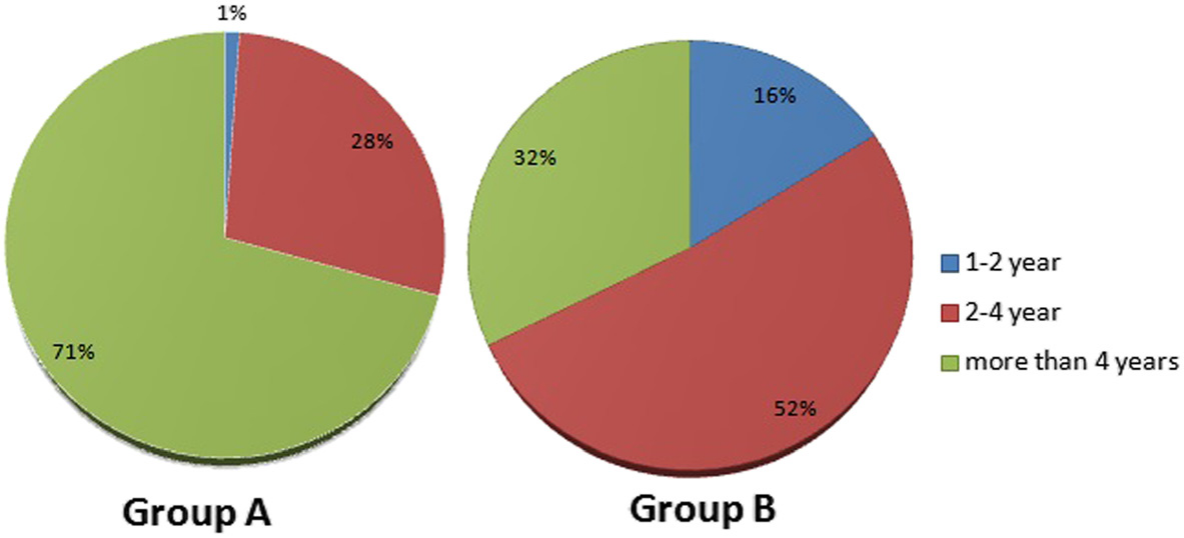
Infertility period in Group A and B participants versus the use of alternative and antioxidant therapies for infertility treatment.

The present study investigated the use of alternative and antioxidant therapy for infertility treatment among males in Jordan. A total of 500 questionnaires were distributed, of which 428 were completed (response rate = 85.6%). Table 1 summarizes the demographic characteristics of the study population. Study participants were mostly middle aged, from 31–45 years (82%, 353/428). Patients were fairly educated, with 41% (175/428) holding bachelor’s degrees. The patient population was of an intermediate economic level, earning 500–1000 JD monthly (50%, 210/418). Most patients had a male factor infertility disease course of more than 2 years (90%, 387/428).Of the 428 infertile men who completed the questionnaire, alternative and antioxidant therapies used for infertility treatment were encountered in 43% of patients (184/428) (Group A). Remarkably, more than two thirds of the participants who used alternative and antioxidant therapies were older than 36 years (Figure 1). The prevalence of alternative and antioxidant therapies considerably decreased in younger ages, as Figure 1 clearly demonstrates. On the other hand, interest in alternative and antioxidant therapies for infertility treatment diminished in older patients (more than 46 years), suggesting a correlation between age and the propensity to use CAM.In the study sample, the tendency to use alternative and antioxidant therapies in Group A participants significantly (p < 0.05) increased as the period of infertility increased. As illustrated in Figure 2, about 71% (130/184) of Group A participants suffered from infertility for more than 4 years, compared with only 32% (79/244) of Group B participants. Infertile males who reported the use of alternative and antioxidant therapies were mostly infertile for more than 4 years.

**Table 1:** Characteristics of infertile males participating in the study at Amman, Jordan 2013 (N = 428)

**Characteristic**	**Number**	**Percentage**
**Age (N = 428)**		
18–25	11	2.6
26–30	32	7.5
31–35	44	10.3
36–40	140	32.7
41–45	169	39.4
Over 46	32	7.5
**Education (N = 428)**		
Primary school	16	3.7
High school	56	13.1
Community college	148	34.6
Undergraduate studies	175	40.9
Postgraduate studies	33	7.7
**Income JD**^**a **^**(N = 418)**^**b**^		
Less than 500 JD	51	12.2
500–1000 JD	210	50.2
1000–1500 JD	93	22.2
1500–2000 JD	36	8.6
More than 2000 JD	28	6.7
**Occupation (N = 414)**^**b**^		
Employee in medical sector	76	18.3
Employee in non medical sector	263	63.5
Business owner	37	8.9
Retired	26	6.3
Unemployed	12	2.9
**Infertility period (N = 428)**		
1–2 years	41	9.6
2–4 years	178	41.6
More than 4 years	209	48.8

^a^Jordanian Dinar (JD 0.71 = USD 1.0).

^b^Total number <428 due to unanswered questions/missing data.

**Figure 3:**
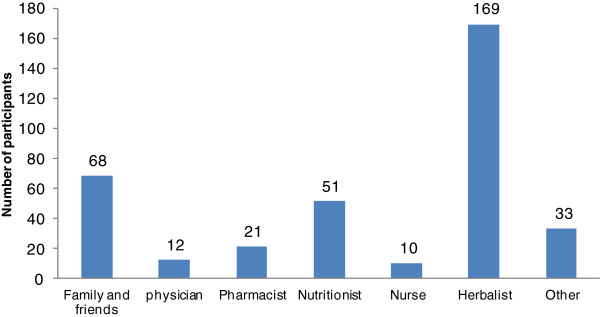
Sources of recommendation for the usage of alternative and antioxidant therapies for infertile male patients in Jordan.

**Figure 4:**
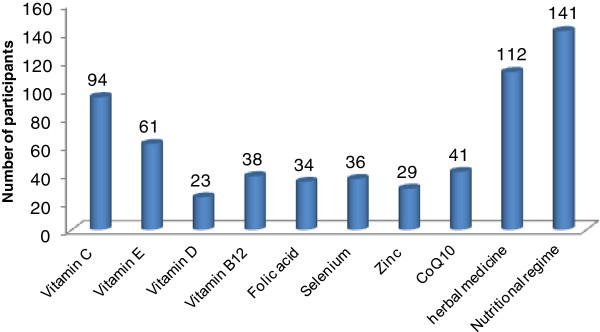
Common alternative and antioxidant therapies used by infertile male patients in Jordan.

Our study population consisted of 18.3% (76/414) infertile males who work in the medical field (e.g. nurses, dentists, pharmacists, physicians). Our analysis clearly demonstrates that this particular category of infertile males was least interested in alternative and antioxidant therapies for their infertility treatment. Indeed, only 2.7% (5/184) of infertile males who work in the medical field reported the use of alternative and antioxidant therapies in their therapy course.According to our results, the main sources for alternative and antioxidant therapy recommendations were herbalists at 92% (169/184), followed by family and friends at 37% (68/184). Nutritionists were also major sources for recommendations, as 28% (51/184) of Group A received recommendations to use alternative and antioxidant therapies from licensed nutritionists. Very few recommendations were made by physicians and pharmacists, as shown in Figure 3.Vitamins, such as C and E, were the most commonly reported antioxidants used by Group A participants. Participants specified that vitamins were used individually and not as multivitamin products. Approximately, 51% (94/184) of Group A participants reported the use of vitamin C, while 33% (61/184) of Group A participants declared the use of vitamin E. Minerals, such as selenium and zinc, were reported by less than 20% of Group A participants (Figure 4). Remarkably, almost 60% (112/184) of Group A participants used herbal medicines for their infertility treatment (Figure 4). Unfortunately, most participants were not clear about the herbal medicines used for their treatment. Participants described these herbal formulations as herbal mixtures provided by herbalists. Nevertheless, 40% (45/112) of participants who reported the use of herbs clearly identified ginger, saw palmetto, and ginseng as the main herbal constituents used in their adjunct treatment course.By and large, nutritional regime appears to be the most common alternative therapy used to treat infertility in Group A participants. Almost 76% (141/184) of Group A participants were on a nutritional regime during their treatment course (Figure 4). Participants described their diet as vitamin-rich with increased protein servings per week. Foods like eggs, red meat, nuts, spinach, fish, butter, and whole fat dairy products were specifically mentioned when participants were asked about their diet. Only 36% (51/141) of the patients obtained their diet from nutritionists, while 64% (90/141) obtained their diet from other sources. Surprisingly, when the participants who consulted nutritionists for their diet were asked whether they reported any vitamin, mineral, or herbal supplement use to their nutritionist, none of the answers were positive.

Likewise, statistical analysis of the response to a question asking whether participants discussed their CAM use, including their diet, with their physicians, revealed that only 10.3% (19/184) of Group A participants declared their CAM use to their health care provider. Surprisingly, these participants asserted that their health care providers neither approved nor denied their alternative and antioxidant therapy use. Participants indicated that their health care providers were neutral towards their CAM use.

**Figure 5:**
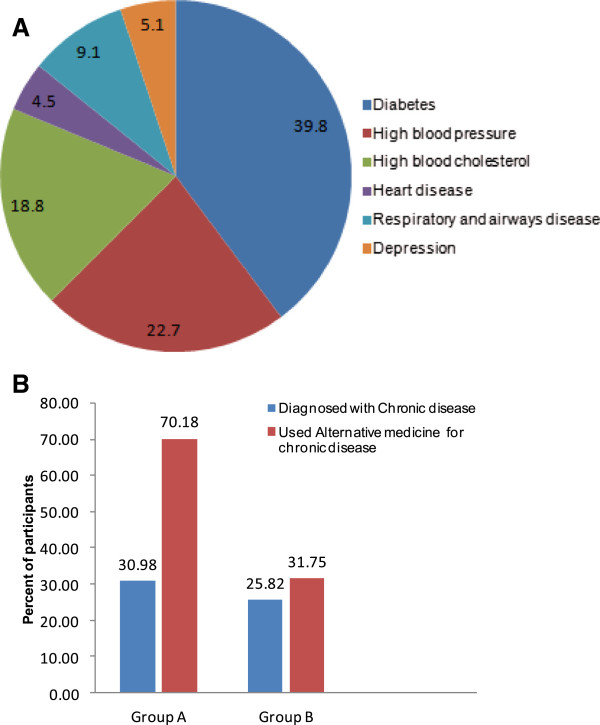
**Alternative and antioxidant therapies utilization by infertile males for the treatment of their chronic conditions. A**. Prevalence of chronic diseases in infertile male patients in Jordan. **B**. Patterns of alternative and antioxidant therapies used for the treatment of other chronic diseases in Groups **A** and **B**.

Our results indicate that, relative to Group B participants, Group A participants trusted CAM therapies to be safe and lack side effects. More than two thirds of Group A participants (145/184) believed in the safety of alternative and antioxidant therapies for the treatment of their infertility. Only 21.2% (39/184) of Group A participants were aware of potential side effects associated with some alternative and antioxidant therapies. In contrast, 61% (149/244) of Group B participants appreciated the possible side effects of CAM therapies.Approximately one third of Group A participants (57/184) and about 26% (63/244) of Group B participants were diagnosed with other chronic medical conditions in addition to their infertility. Both diabetes and high blood pressure were the most commonly encountered chronic diseases in the study sample (Figure 5A). Interestingly, the tendency to use CAM therapies for chronic medical conditions appears to be highly correlated to their use for infertility treatment. Indeed, 70.1% (40/57) of Group A participants who were diagnosed with other chronic medical conditions reported the use of alternative therapies for their medical condition treatment (Figure 5B). On the other hand, only 31.8% (20/63) of Group B participants who were diagnosed with other chronic medical conditions reported the use of alternative therapies for their medical condition treatment (Figure 5B).

**Figure 6:**
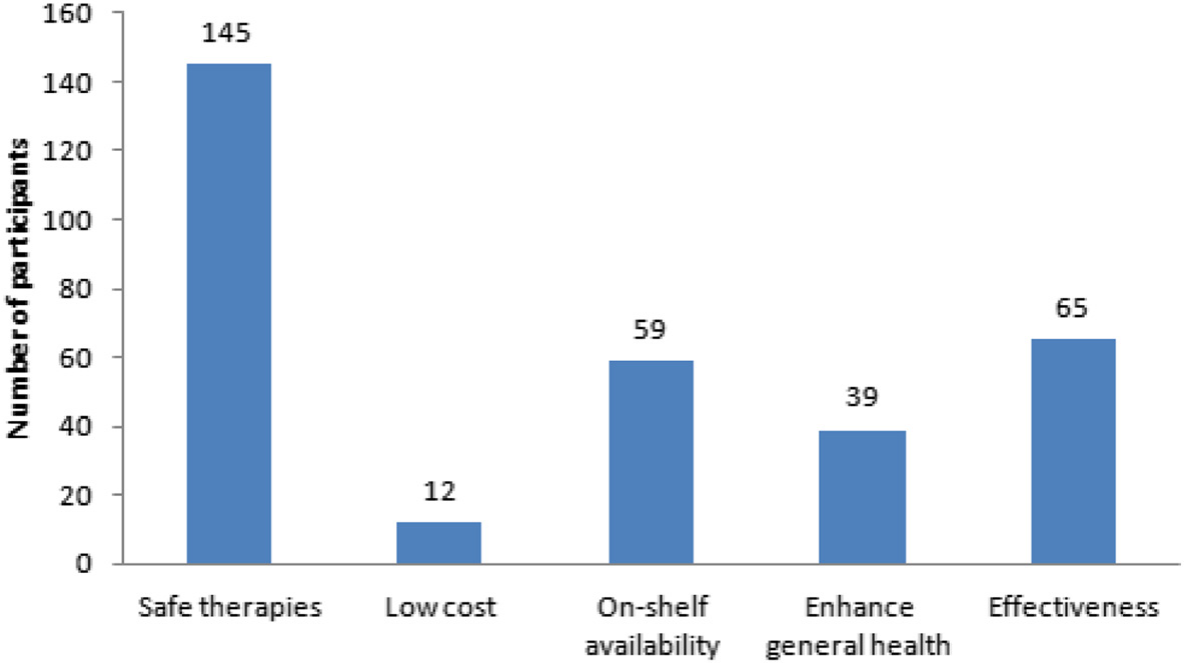
Motives for alternative and antioxidant therapy use for infertility treatment in infertile male patients in Jordan.

When Group A participants were asked whether the alternative and antioxidant therapies used for their infertility were actually helpful to enhance their fertility, only 17% (31/184) of Group A participants reported positive outcomes. However, only 6.5% (2/31) of those participants could relate the reported positive outcomes to increased pregnancy rate. Rather, 93.5% (29/31) of the participants related the reported positive outcomes associated with their use of alternative and antioxidant therapies to general health status enhancement.Despite the lack of evidence on the effectiveness of alternative and antioxidant therapies for infertility treatment, Group A participants generally related their use of such therapies to effectiveness, on-shelf availability, low cost, and high safety (Figure 6). Often, Group A participants believed that if alternative and antioxidant therapies are not useful, then they are also not harmful. While there was a reported low efficacy of alternative and antioxidant therapies to treat infertility, a significant number of Group A participants believed that their use of CAM therapies was effective (Figure 6). This belief in effectiveness was not supported by any evidence related to increased pregnancy rates.

## Discussion

Male infertility is a multi-faceted condition with several potential contributing factors. Most commonly, occupational, environmental, and nutritional factors contribute to sperm count and motility. Sperm count and motility are ultimately responsible for fertility. Spermatogenesis is an energetic process that demands an optimal intake of antioxidants, minerals, and nutrients. Disruption of the balance between these nutrients is suggested as one of the possible etiologies of male infertility [8].

ROS, such as the superoxide anion, hydrogen peroxide, and nitric oxide, are needed for fertilization, and thus are produced by spermatozoa. Nevertheless, high concentrations of these free radicals can directly damage sperm cells [8], resulting in male infertility. Antioxidants are the first line of defense against ROS-induced male infertility. Numerous clinical trials have investigated the potential role of antioxidant therapy to relieve ROS-induced male factor infertility [23]. Although antioxidants are hypothesized to be useful therapeutic agents for male infertility, the results of clinical trials are controversial [23,24]. In this study, we investigated the patterns of antioxidant use among infertile male patients in Jordan. Additionally, the use of alternative therapies, including herbal products and nutritional regime, were evaluated in a population of 428 infertile males in Jordan.

The human body employs three general tactics of antioxidants to safeguard against free radicals: endogenous antioxidants, dietary antioxidants, and metal-binding proteins [25]. Low molecular weight molecules, such as coenzyme Q-10, and larger molecular enzymes, such as superoxide dismutase, catalase, and glutathione peroxidase, are classified as endogenous antioxidants [25]. Vitamins C and E, carotenoids, and flavonoids are classified as dietary antioxidants that act through scavenging free radicals to break the chain reaction responsible for lipid peroxidation. This class of antioxidants is known as chain-breaking antioxidants [25]. On the other hand, metal-binding proteins, such as albumin, ferritin, and myoglobin, inactivate the transition metal ions that catalyze the production of free radicals [26,27].

In this study sample, the use of chain-breaking antioxidants, such as vitamin C and vitamin E, was more frequently encountered relative to other antioxidants (Figure 4). Vitamin C (ascorbic acid) is a water-soluble ROS scavenger, while vitamin E is an important lipid-soluble antioxidant. The hydrophilicity and lipophilicity of vitamins C and E may function synergistically to scavenge ROS and protect spermatozoa. Although no decisive conclusion has been confirmed in regard to the beneficial role of vitamins C and E in male infertility [23], some reports suggest that sperm motility and characteristics deteriorated significantly when a combination of vitamins C and E was used [23,28,29].

Additionally, our study demonstrates that a significant percentage of men presenting for infertility assessment have used herbs as adjunct therapies to enhance their fertility (Figure 4). The potential of herbs to enhance male fertility has not been extensively studied [30,31]. According to our results, ginger is one of the main herbal ingredients used by Group A participants. Ginger extracts have been widely studied for an extensive scope of biological activities, especially antioxidant activities [32]. In rats, ginger considerably reduced lipid peroxidation by sustaining the activities of antioxidant enzymes superoxide dismutase, catalase, and glutathione peroxides [32]. Saw palmetto was also one of the main herbs used to treat infertility among Group A participants. Recently, the use of saw palmetto for the treatment of benign prostatic hyperplasia and lower urinary tract symptoms in males has increased [33,34]. Nevertheless, the precise mechanism of action of saw palmetto remains unknown.

Recent studies suggest several beneficial effects of ginseng to increase the antioxidant capacity of various tissues [35]. Both Asian and American ginseng were shown to enhance libido and copulatory performance in laboratory animals [36,37]. The observed effects of ginseng are possibly related to its ginsenoside components on the central nervous system and gonadal tissues [36,37]. In addition, ginsenosides were shown to increase sperm motility [38] and facilitate erectile dysfunction in males [39].

One of the main points addressed by our analysis is the poor communication between the patients and their health care providers. Only 10.3% (19/184) of Group A participants declared their alternative and antioxidant therapies to their physicians. Increased consumption of certain antioxidants, regardless of their source, with the concurrent use of herbal products rich in antioxidants has been suggested to significantly deteriorate sperm motility and characteristics [28,29]. Although our study design was unable to establish a negative or positive correlation between the use of alternative and antioxidant therapies and an increase in pregnancy rate, the increased prevalence of alternative and antioxidant therapies among infertile males necessitates a clear and urgent need for physicians to become more aware of this phenomenon and conduct further research in this field [23].

## Conclusions

This study provides results of a survey of infertile males in Jordan (n = 428) seeking medical assistance for their infertility. Our data show that the use of alternative and antioxidant therapies for infertility treatment is more common in middle-aged and fairly educated infertile males who have suffered from infertility for more than 4 years. Nutritional regime, herbal formulations, and vitamins such as vitamin C and E were the most popular alternative and antioxidant therapies used in the study population. Our results indicate that the majority of infertile males who used alternative and antioxidant therapies did not inform their health care providers about their adjunct therapies. Some of these adjunct treatments might have adverse effects on the outcomes of conventional medical treatment. Therefore, it is essential to inquire about the use of these therapies by infertile males presenting at infertility clinics.

## Competing interests

The author declares that he has no competing interests.

## Pre-publication history

The pre-publication history for this paper can be accessed here:

http://www.biomedcentral.com/1472-6882/14/244/prepub

## Supplementary Material

Additional file 1 Alternative and antioxidant therapies utilization by a sample of infertile males in Jordan: a cross sectional study. Click here for file
