# Supplementary material for: Alternative and antioxidant therapies used by a sample of infertile males in Jordan: a cross-sectional survey
Source: BMC Complement Altern Med. 2014 Jul 16;14:244. doi: 10.1186/1472-6882-14-244 (PMC4223394; doi:10.1186/1472-6882-14-244)
Supplement: Additional file 1 — Alternative and antioxidant therapies utilization by a sample of infertile males in Jordan: a cross sectional study. [file 1472-6882-14-244-S1.doc]

**Alternative and antioxidant therapies utilization by a sample of infertile males in Jordan: a cross sectional study**

**Form Number:**

**April 2013 - September 2013**

Any information collected through surveys will be used in reports only on an aggregate basis.  That is, it will not be possible for anyone to identify a particular individual with any set of responses. We strive to ensure that data is kept secure, and that we collect only as much personal data as is required for the survey. We assure you that your responses will be kept confidential.

**Please ticks (X) in the appropriate answer box □or fill in the provided space for the following questions**:

1. **Age** □ 18-25 □ 26-30 □ 31-35 □ 36-40 □41-45 □over 46
2. **Education level** □ Primary school □High school □Community college

□Undergraduate studies □Postgraduate studies

1. **Occupation □** Employee in medical sector (physician, dentist, pharmacist, nurse…etc) **□** Employee in non medical sector **□** Business owner **□**Retired **□**Unemployed **□** Other (specify please…………………………………………………………………..)

1. **Monthly income of the family** □Less than 500 JD □500-1000 JD □1000-1500 JD

□1500-2000 JD □ More than 2000 JD

1. **Do you have an infertility condition, if yes, for how long have you been suffering from infertility?**

□1-2 years □ 2-4 years □More than 4 years

1. **Have you ever been diagnosed with any chronic medical condition?**

□Yes □No

**If your answer to Q6 was Yes, please answer Q7-8; if NO, please move to Q9*

1. **Which of the following chronic medical conditions have you been diagnosed with?**

□Diabetes □ High blood pressure □ High blood cholesterol □Heart disease □Respiratory and airways disease □ Depression

□ Other ( specify……….)

1. **Have you ever used complementary and alternative therapies (CAM) for the treatment of your chronic medical conditions?**

□Yes □No

1. **Have you ever used CAM therapies for the treatment of your infertility condition?**

□Yes □No

1. **If you used CAM therapies for infertility treatment, then where did you get the recommendations to use such therapies?**

□Family and friends □ physician □Pharmacist □Nutritionist □Nurse □Herbalist □Other (mention…………………….)

1. **If you used CAM therapies for infertility treatment, then which of the following regimens did you use?**

□Vitamin C □ Vitamin E □ Vitamin D □ Vitamin B12

□Selenium □Folic acid □CoQ10 □Zinc

□Multivitamins products (please name the product…………………………….. )

□Nutritional regime (Please describe …………………………………………….)

□Herbs from the herbalist (please name the herbs…………………………………….…)

□ Other (please specify……………………………………………………………)

**If you were on a nutritional regime for infertility treatment, please answer Q12-13, if no, please proceed to Q14*

1. **If you were on a nutritional regime for infertility treatment, where did you get the regime from:**

□Nutritionist □ Other sources

1. **If you were on a nutritional regime for infertility treatment AND used other CAM therapies (herbs, vitamins, minerals), did you inform the nutritionist about the CAM therapies used:**

□Yes □No

1. **If you used CAM therapies, including herbs, vitamins, minerals and nutritional regime, did you inform your physician about the used therapies?**

□ Yes (*If yes, please specify if the physician approved the utilization of CAM therapies…………………………………….………*)

□No

1. **If you used CAM therapies, including herbs, vitamins, minerals and nutritional regime, for infertility treatment, your reasons for the utilization of such therapies could be:**

**□**CAM are safe therapies □ CAM has low cost □ CAM is easily available

□ CAM is effective to treat infertility

□CAM is good to general health

1. **If you used CAM therapies, including herbs, vitamins, minerals and nutritional regime, for infertility treatment, was the therapies used for your infertility condition helpful?**

□Yes □No

1. **If the answer to Q16 was Yes, then was the CAM therapy used helpful in:**

□ General health enhancement □Increasing pregnancy rate in your partner

□ Others ( specify…………………………………………………….)

1. **Do you believe that herbs, vitamins, minerals and other forms of CAM therapies are safe to be used for infertility treatment?**

**□**Yes □No
